# Predictors of Mortality in Medical ICU Patients: A Retrospective Study in a Tertiary Care Center in Jordan

**DOI:** 10.3390/jcm14124039

**Published:** 2025-06-07

**Authors:** Tarek Gharibeh, Munir Abu-Helalah, Hussam Alshraideh, Manar Abu Awwad, Zaid Al Bzour, Majd Abuzayed, Luma Taweel, Zahraa Al-Fayyadh, Bushra Wraikat, Yomna Alfaqeeh, Layan Aburumman

**Affiliations:** 1 Department of Internal Medicine, Faculty of Medicine, University of Jordan, Amman 11942, Jordan; manarabuawwad6@gmail.com (M.A.A.); zaid.bzour@yahoo.com (Z.A.B.); majd.zaid1995@gmail.com (M.A.); lumataweel@hotmail.com (L.T.); alizhra@icloud.com (Z.A.-F.); bushrawraikatfaysal@gmail.com (B.W.); yomnaalfaqeeh@gmail.com (Y.A.); layan1.aburumman@gmail.com (L.A.); 2 Department of Family and Community Medicine, Faculty of Medicine, University of Jordan, Amman 11942, Jordan; m.abu-helalah@ju.edu.jo; 3 Public Health Institute, University of Jordan, Amman 11942, Jordan; 4 Department of Industrial Engineering, American University of Sharjah, Sharjah P.O. Box 26666, United Arab Emirates; halshraideh@aus.edu; 5 Industrial Engineering Department, Jordan University of Science and Technology, Irbid 22110, Jordan

**Keywords:** ICU, mortality, predictors

## Abstract

**Background/Objectives**: This study aims to investigate ICU mortality rates and to identify predictors of ICU mortality, focusing on clinical and demographic variables, including age, comorbidities, hemoglobin and creatinine values, intubation in the Emergency department, and Glasgow Coma Scale (GCS) and APACHE II scores at presentation in the Emergency department, and how these factors influence patients’ clinical outcomes. **Methods**: This retrospective observational cross-sectional study analyzed patients admitted to the Jordan University Hospital (JUH) ICU from 1 January 2022 to 31 December 2023. A total of 1323 patients were included, with a mean age of 65 ± 17 years, of whom 442 (34%) died during their ICU stay. **Results**: A delay of 6 h or more in ICU admission was reported for 77% of the participants. Mortality rates were significantly lower among patients admitted to the ICU through the Emergency department (32%) compared to those transferred from other wards (41%) (*p* = 0.003). Higher mortality rates were observed among patients on vasopressors and those intubated in the Emergency department, with lower median hemoglobin (Hb) levels, higher APACHE II scores, and pneumonia as the main diagnosis or urosepsis as the secondary diagnosis (*p* < 0.001). **Conclusions**: This study identified predictors of mortality in a medical ICU at a tertiary hospital in Jordan.

## 1. Introduction

The Intensive Care Unit (ICU) is a high-stress and time-sensitive environment where patients are often in critical condition and have a high risk of mortality [1,2]. Predicting ICU outcomes can help in decision-making for healthcare providers in selecting treatment plans, as well as the patients and their families [3]. ICU mortality remains the most important outcome and the main concern of many studies; various factors have been explored, such as age, demographics, delayed admissions, creatinine level, and Glasgow Coma Scale [1,4,5,6]. A retrospective multicenter cohort study analyzing data from 3885 health records of patients who died between January 2014 and January 2017 across eight medical ICUs in Jordan found a high mortality rate of 34.6%, with a slight decline observed from 2014 through 2016. Among the deceased, 46.8% were transferred from the Emergency department, and 74% had multiple comorbidities [7].

Various tools have been adopted to improve outcomes in predicting ICU mortality and morbidity. One of the most widely used scoring systems in adult ICUs worldwide is the Acute Physiology and Chronic Health Evaluation II (APACHE II) [8]. APACHE II provides a general measure of the severity of the disease, using a numerical score based upon the initial values of 12 routine physiologic measurements, age, and previous health status [9]. This tool translates clinical data into a numeric expression to predict the illness severity and estimate the mortality rate for each patient [10].

Several studies have explored the use of the APACHE score to predict mortality and hospital length of stay across various diagnoses [11,12,13]. The sensitivity and specificity of the score varied depending on the condition, with values reported as 100% and 30%, respectively, in surgical Intensive Care Units [14], and 87% and 54% in patients with breast cancer [15]. However, no study has investigated differences in APACHE score performance across different diagnoses within the same institution. Additionally, there is no available data on ICU admission reasons or delays in admission specific to Jordan.

Studies exploring the correlation between anemia and clinical outcomes have yielded inconsistent results. A meta-analysis found that ICU patients with anemia were more prone to developing acute kidney injury (AKI). Subgroup analyses demonstrated that anemia was linked to increased all-cause mortality among patients with AKI, trauma, cancer, sepsis, and those in cardiac ICUs. However, this association was not evident in patients with traumatic brain injury [16].

Accident and Emergency department (Emergency department) crowding is an escalating global challenge, particularly for critically ill patients. This has led to Emergency department boarding, defined as the inability to admit critically ill patients to the Intensive Care Unit (ICU) due to the lack of ICU beds [17]. Emergency department boarding has been associated with adverse patient outcomes [18,19].

Admission delays are common; in addition to the logistical reasons, there are triage and diagnostics reasons [4]. Prolonged hospital length of stay (LOS) prior to the admission of patients from wards to the ICU is associated with an increased mortality rate [20]. On the other hand, it has been shown that direct ICU admission from the Emergency department has decreased 30-day mortality compared to admission to the ICU within 24 h of ward admission [5].

Numerous studies have linked Emergency department boarding to poorer outcomes in patients requiring ICU admission [21,22,23,24], although the findings are not entirely consistent. Some studies report prolonged lengths of stay (LOSs) [19], while others find no significant changes [18,25]. Similarly, hospital mortality results are mixed, with some studies noting an increase in mortality [21,22] and others reporting no significant difference [24]. Additional outcomes include an increased risk of organ dysfunction [23] and a greater likelihood of requiring mechanical ventilation [18]. Groenland et al. (2019) proposed that patient diagnosis might play a role in these outcomes, citing higher hospital mortality rates associated with cardiac arrest [4].

We conducted a retrospective single-center observational cross-sectional study on critically ill patients admitted to the medical ICU at Jordan University Hospital (JUH) to investigate mortality rates and predictors of ICU mortality at our hospital.

## 2. Materials and Methods

### 2.1. Study Design and Setting

We conducted a retrospective observational cross-sectional study of patients who were admitted to our Intensive Care Unit (ICU) at Jordan University Hospital (JUH) from the 1 of January 2022 to the 31 December 2023. Jordan University Hospital is a 600-bed tertiary teaching hospital located in Amman, Jordan. JUH encompasses all major and sub-medical and clinical specialties, containing 64 different specialties. Our medical ICU has a capacity of 20 beds, with a 2:1 nurse-to-patient ratio. Data were collected using JUH electronic medical records from the 24 of March 2024 to the 9 of December 2024 by internal medicine residents at JUH and 3 senior medical students at the University of Jordan.

The primary outcome of this study was measuring the ICU mortality, while the secondary outcomes included investigating predictors of mortality, focusing on clinical and demographic variables including age, comorbidities, hemoglobin and creatinine values, intubation in the Emergency department, Glasgow Coma Scale (GCS) scores, and APACHE II scores at presentation in the Emergency department, and how these factors influence patients’ clinical outcomes. Another important secondary outcome of this study was to evaluate whether Emergency department boarding to the ICU was associated with higher mortality rates compared to ward-to-ICU admission and to explore the impact of the duration of ICU admission delay and the length of ICU stay on patients’ survival.

### 2.2. Study Population

Our study included 1323 patients of both sexes who were admitted to the ICU at JUH. The inclusion criteria were as follows: (1) patients who were aged 18 years or older, (2) patients who were hospitalized from January 2022 to December 2023, (3) patients who were admitted specifically to the medical ICU, and (4) patients with available clinical records. Patients who were admitted to the surgical ICU, cardiac ICU, or neurological ICU were excluded from the study. According to the route of admission, patients were divided into two categories: patients who were admitted directly from the Accident and Emergency department (Emergency department) and patients who were transferred from inpatient wards.

The following information was collected manually from the electronic records. TG and MA double-checked collected data through random sample evaluation.

ICU mortality: The primary outcome of the study.

- Patient demographics: Age, gender.
- Comorbidities: Diabetes mellitus, Hypertension, Chronic Kidney Disease, liver cirrhosis, Ischemic Heart Disease, Heart Failure, chronic obstructive pulmonary disease, Asthma, obstructive sleep apnea.
- Date of Emergency department and ICU admission.
- ICU stay duration.
- Primary and secondary diagnosis: Respiratory Failure Type 1, “also known as Hypoxemic Respiratory Failure, which defined as inadequate oxygenation of hemoglobin”; Respiratory Failure Type 2, “also known as Hypercapnic Respiratory Failure, it occurs when alveolar ventilation is inadequate to clear CO_2_ produced by cellular metabolism and the level of CO_2_ increases in blood”; pneumonia (hospital-acquired pneumonia or community-acquired pneumonia); Stroke; Non-ST Elevation Myocardial Infarction; Upper Gastrointestinal Bleeding; acute kidney injury; decompensated liver cirrhosis, “It’s defined as patient with liver cirrhosis who have developed complications of cirrhosis, such as variceal hemorrhage, ascites, spontaneous bacterial peritonitis, hepatocellular carcinoma, hepatorenal syndrome, or hepatopulmonary syndrome”; Diabetic Ketoacidosis; Acute Respiratory Distress Syndrome, “Which defined as is an acute, diffuse, inflammatory form of lung injury that is associated with a variety of etiologies”; Acute Decompensated Heart Failure, “It’s a clinical syndrome of new or worsening signs and symptoms of HF that often lead to hospitalization or an emergency department visit”; urosepsis, “It’s a life-threatening organ dysfunction caused by a dysregulated host response to urinary tract infection”; Acute Liver Failure, “which defined as acute liver injury, hepatic encephalopathy (altered mental status), and an elevated prothrombin time/international normalized ratio (INR)”; Severe Asthma Exacerbation; Acute Pancreatitis; and others.
- Administration of vasopressors in the Emergency department.
- Whether the patient was intubated before ICU admission.

The following variables were collected on the day of Emergency department admission:

- Glasgow Coma Scale (GCS);
- Initial laboratory findings (hemoglobin and creatinine levels);
- Acute Physiology;
- Chronic Health Evaluation (APACHE) II score.

### 2.3. Ethical Approval

This study was approved by the scientific committee and the Institutional Review Board (IRB) at Jordan University Hospital (10120251809). The Ethics Committee waived the requirement for informed consent from patients due to the retrospective and observational nature of the study, as well as the anonymization of the data.

**Sample size calculations:**

Based on the expected mortality rates in developing countries, a conservative estimate of 30% was used. A sample of 1291 was required, with a 95% confidence interval and a margin of error of 0.025 [26].

### 2.4. Statistical Analysis

Mortality rates were calculated based on the proportion of patients who died during the study duration of two years. Data are presented as a median with a standard deviation (SD). Patients were dichotomized based on survival at ICU discharge. The Wilcoxon rank-sum test and Pearson’s Chi-squared test were used to analyze data based on the admission source (medical wards vs. emergency services). Three logistic regression models were performed. In the first model, the APACHE score was used to predict mortality while dropping features that contribute to the APACHE score such as age and GCS. In the second model, the APACHE score was dropped, and all remaining features were used to predict mortality including age, GCS, and Cr. Model 3 predicts mortality through all features set including APACHE, age, Cr, and GCS. The point of adding model 3 is to compare the model performance with the other two models and identify any potential collinearity. Where applicable, missing values were imputed through the median value as a robust statistic.

## 3. Results

During the period between January 2022 and December 2023, 1323 participants fulfilled our inclusion criteria. Data for 20 patients were dropped, as they had a length of stay in the ICU for more than 1000 h, to avoid any possible bias in the analysis. A total of 76% of the patients were admitted through the Emergency department, while the rest were transferred from inpatient wards.

As shown in Table A1, the mean age of study participants was 65 ± 17 years, of which 442 (34%) died during their stay in the ICU. Fifty-three percent of patients were males, and 77% had a delay in admission of 6 h or more. The most common disease among all patients was HTN (60%), followed by DM (51%). On the other hand, the most common main diagnosis was pneumonia (21%).

The median delay in admission was 12 h (SD: 15 h), with statistically significant longer delays in admissions through the Emergency department when compared with our routes (15 vs. 7, *p* < 001). This was reflected in the proportion of patients with admission delay (>6 h), as indicated in Table 1. Of those patients admitted through the Emergency department, 83% had a more than 6 h delay, while 17% of those admitted through other routes had a delay of more than 6 h (*p* < 0.001). There was a statistically significant difference in the mortality rates between these two groups, with a lower mortality rate of 32% for admissions through the Emergency department compared with 41% for patients transferred to the ICU from other wards (*p* = 0.003). In terms of comorbidities, there was a statistically significant difference between patients admitted through the Emergency department and those from other wards in the prevalence of HTN (79% vs. 21%, *p* = 0.010). Other medical history and clinical characteristics of participants by route of admission are shown in Table A1.

Table 1 describes the baseline characteristics of participants grouped by outcome at discharge. The age of patients with death as an outcome of admission was significantly higher than those who was discharged alive (69 years vs. 63 years, *p* < 0.001). Expectedly, they had a lower mean GCS (12.78 vs. 14.38, *p* <0.001) at admission. This trend was also seen with the median duration of ICU stay, which was also higher for those who died (173 h vs. 80 h, *p* < 0.001). Furthermore, those diagnosed with COPD/Asthma had lower mortality rates than those who were not (27% vs. 35%, *p* = 0.041), and those diagnosed with OSA had the same trend (22% vs. 35%, *p* = 0.026). On the contrary, those diagnosed with HAP had higher mortality rates (49%) than both those diagnosed with CAP (46%) and those not diagnosed with pneumonia (30%) (*p* < 0.001).

Participants on vasopressors had a higher mortality rate than those not on vasopressors (53% vs. 29%, *p* < 0.001). The same was seen in intubated patients compared with patients not intubated (77% vs. 30%, *p* < 0.001). Moreover, patients who died had lower median Hb levels (10.80 vs. 11.50, *p* < 0.001), but a higher median APACHE score (18 vs. 15, *p* < 0.001). Participants with pneumonia being their main diagnosis had significantly higher mortality rates (49% vs. 30%, *p* < 0.001). Lastly, participants diagnosed with urosepsis as their secondary diagnosis had higher mortality rates than those without this secondary diagnosis (64% vs. 33%, *p* < 0.001) (Table 1).

The logistic regression in Table 2 shows predictors of mortality by including the APACHE score only and dropping other features that contribute to the APACHE score, namely, age, GCS, and Cr. Those admitted through the Emergency department had lower odds of mortality (*p* = 0.007). Similarly, diagnosis with CKD (*p* = 0.1), COPD/Asthma (*p* = 0.017), and a higher Hb level (*p* = 0.001) lowered the odds of mortality. On the other hand, being on vasopressors (*p* < 0.001), intubated (*p* < 0.001), having a main diagnosis of pneumonia (*p* = 0.002), Stroke (*p* = 0.079), NSTEMI (*p* = 0.11), and having a secondary diagnosis of urosepsis (*p* = 0.009) all increased the odds of mortality. As expected, a higher APACHE score indicates a higher probability of death (*p* < 0.001). The model performance in predicting mortality was also assessed in this study. The model’s sensitivity was 89.2%, specificity was 42.08%, accuracy was 73.22%, area under ROC curve (AUROC) was 0.78, and Brier score was 0.17.

Logistic regression in Table 3 examined the predictors of mortality without the APACHE score as the model input. The results were similar to those in Table 2. Patients admitted through the ER had lower odds of mortality (*p* = 0.003). A higher GCS (*p* < 0.001) and higher Hb level (*p* = 0.001) lowered the odds of mortality. On the other hand, a higher age (*p* < 0.001), being on vasopressors (*p* < 0.001), intubated (*p* < 0.001), having a main diagnosis of pneumonia (*p* = 0.002), and having a secondary diagnosis of urosepsis (*p* = 0.027) all increased the odds of mortality. The model’s sensitivity was 88.97%, specificity was 45.02%, accuracy was 74.06%, AUROC was 0.79, and Brier score was 0.17.

Table 4 shows the predictors of mortality with the APACHE score and all of its contributing factors including age, GCS, and Cr. The results were similar to those in Table 2 and Table 3. Patients admitted through the ER had lower odds of mortality (*p* = 0.004). A higher GCS (*p* < 0.001) and higher Hb level (*p* = 0.001) lowered the odds of mortality. On the other hand, being on vasopressors (*p* < 0.001), intubated (*p* < 0.001), having a main diagnosis of pneumonia (*p* = 0.002), higher APACHE score (*p* = 0.018), and having a secondary diagnosis of urosepsis (*p* = 0.029) all increased the odds of mortality. The model’s sensitivity was 89.02%, specificity was 44.8%, accuracy was 74.14%, AUROC was 0.79, and Brier score was 0.17.

**Figure A1:**
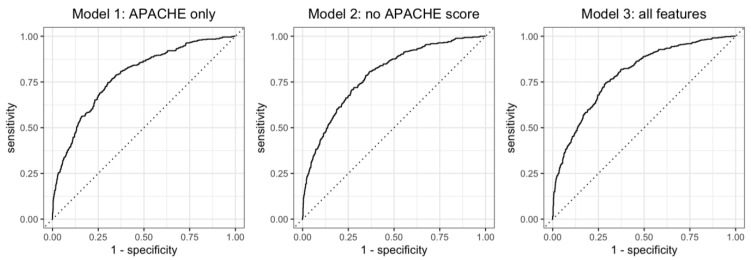
ROC curves for the three logistic regression models fitted. Solid lines represent the fitted model ROC curve while the dotted diagonal line represents the ROC curve of a random guess mod-el. For all cases, the fitted model ROC curve dominates the random guess model curve showing an improved performance with AUROC of 0.78, 079 and 0.79, respectively.

**Figure A2:**
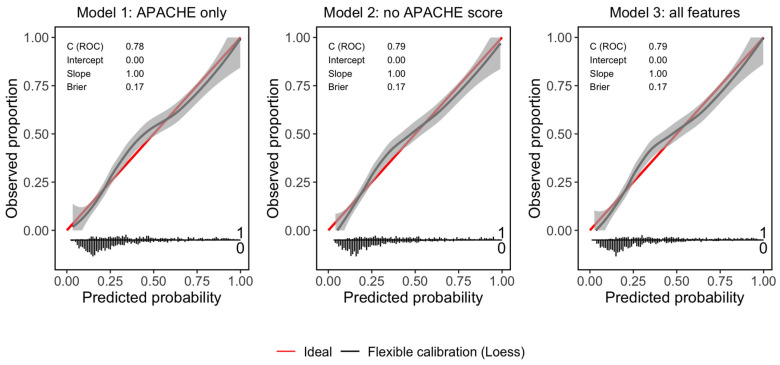
Model calibration plots for the three logistic regression models fitted. Solid red lines represent the ideally calibrated prediction models while the solid black lines show the calibration curve of the fitted prediction models through the Loess smoothing method. Shaded grey areas rep-resent the 95% confidence intervals of the fitted calibration curves.

A performance summary of these three logistic regression models is provided in Appendix A Table A2. The summary shows that all three models performed similarly, with slightly better accuracy provided by the third model, in which all features were used to predict mortality. ROC curves for the fitted models are provided in Appendix A Figure A1, showing the acceptable prediction power of all three models. The results in Table A2 and Figure A1 indicate no multicollinearity issues present in the analysis. The models’ prediction reliabilities were assessed through the Brier score and probability calibration plots provided in Appendix A Figure A2 and Table A2. All models had a Brier score of 0.17, indicating excellent model calibration. Similarly, the calibration curves, shown in Figure A2, indicate perfectly calibrated models.

## 4. Discussion

This is the first study in Jordan to examine predictors of mortality in the ICU. This study found a high ICU mortality rate of 34%, which is higher than rates reported in ICUs of developed countries and comparable with developing countries. A study from Africa reported a mortality rate of 29% [27]; however, it had a smaller sample size and included trauma patients. Similarly, a study from Turkey reported a higher mortality rate of 55%, but the patient population had a higher average age (77 years) and a lower sample size (111 participants) [28]. In contrast, a multicenter study in Europe reported a lower mortality rate of 19% [29]. Although the mean age in the European study is much higher than ours, they mortality rate is half of ours. This indicates the need for improving mortality outcomes in Jordan and other developing countries. The reported discrepancies in mortality rates can be attributed to differences in patient age, comorbidities, and advancements in healthcare systems in developed countries. In Jordan, two studies have reported ICU mortality rates, but both were limited to COVID-19 patients, with mortality rates of 23% and 33% [30,31]. Our data, collected after the pandemic, provide a broader perspective by identifying ICU mortality predictors beyond the COVID-19 era.

Several predictors of mortality in medical ICU patients have been identified, with delayed admission being a significant factor. As a tertiary care center, the University Hospital of Jordan operates a busy ICU, where 77% of patients experience admission delays exceeding six hours, with a median delay of 12 h. This delay is associated with a higher mortality rate. Multiple studies have examined this issue. One study attributed 30% of ICU mortality to admission delays, reporting a 1.5% increase in mortality for every additional hour of delay [29]. Additionally, a large meta-analysis encompassing over 300,000 patients found that delayed ICU admission was associated with a 61% increased risk of mortality (pooled OR: 1.61) [32]. This indicates that more work is needed to reduce this delay in admissions in Jordan.

In this study, the duration of ICU stay has been identified as a predictor of increased mortality. Patients with prolonged ICU stays often have multiple acute conditions, making them more vulnerable to various risks that further elevate mortality rates. These risks include hospital-acquired infections, bedsores, and complications related to prolonged mechanical ventilation. Moreover, extended ICU stays have been linked to higher long-term mortality due to lasting physical, cognitive, and psychological sequelae [33,34].

Similarly, patients with a low Glasgow Coma Scale (GCS) score have lower survival rates, as altered mental status is often associated with high-mortality conditions such as post-cardiac arrest and sepsis. This finding has been confirmed in a large study involving approximately 15,000 patients, where a low GCS score was linked to poor outcomes [35]. Due to its prognostic significance, the GCS was incorporated into the APACHE scoring system, enhancing its predictive accuracy. Additionally, patients requiring mechanical ventilation have higher mortality rates, not only due to the severity of their initial condition but also because of complications associated with prolonged ventilatory support.

The impact of vasopressor use on ICU mortality remains a subject of debate. A large meta-analysis conducted in Italy found that the use of inotropes and vasopressors improved outcomes in patients with septic and vasoplegic shock [36]. However, another study focusing on COVID-19 patients reported a high mortality rate of 69% among those receiving vasopressors, highlighting the variability in outcomes depending on the underlying condition [37]. In our study, the use of vasopressors was associated with increased mortality, likely reflecting the severity of the underlying illness. Patients requiring vasopressor support often present with critical conditions, which inherently carry a higher risk of poor outcomes.

Studies exploring the correlation between anemia and clinical outcomes have yielded inconsistent results. A meta-analysis found that ICU patients with anemia were more prone to developing acute kidney injury (AKI). Subgroup analyses demonstrated that anemia was linked to increased all-cause mortality among patients with AKI, trauma, cancer, sepsis, and those in cardiac ICUs. However, this association was not evident in patients with traumatic brain injury [16].

Anemia is common among ICU patients [38,39], with studies indicating that approximately two-thirds of critically ill patients have a hemoglobin concentration below 12 g/dL [40,41]. Low hemoglobin levels lead to reduced oxygen delivery, which contributes to the poor outcomes associated with anemia. In our study, anemia was linked to higher mortality, a finding consistent with several other studies. This suggests that more clinical work is needed to manage anemia and reduce the associated risk of mortality.

This study found that patients admitted to the ICU from medical wards had higher mortality rates than those admitted from the Accident and Emergency department (Emergency department)—a novel finding that has not been previously reported. This may reflect the complexity of diagnoses and the severity of underlying conditions in ward-admitted patients. Additionally, patients with higher APACHE scores had increased mortality, which aligns with the expectation that sicker patients are at greater risk.

This study is the first of its kind in Jordan. It revealed important clinical quality findings that are generalizable to a wide range of medical conditions, such as delays in transfers and the presence of anemia. Hospital-acquired pneumonia and urosepsis need to be targeted through the local infection control committee. However, this study has several limitations. Firstly, it included data from one center, which limited the external validity. It excluded patients who were admitted to the surgical ICU, cardiac ICU, or neurological ICU. The purpose of this study was to assess medical ICU mortality and we will work on other ICUs in future work. This study included a broad spectrum of medical conditions, which need further assessment separately, and to include comparisons for patients discharged alive with those discharge dead from each condition. There were no follow-ups on survival or outcomes until hospital discharge. Finally, there are unmeasured confounders (e.g., sepsis severity scores and ventilator days).

## 5. Conclusions

This study revealed a high mortality rate at the ICU in our setting when compared with developed countries. This study also identified predictors of ICU mortality in our hospital as part of a quality improvement effort to enhance survival rates at Jordan University Hospital and address existing challenges. While the retrospective nature of the study limits our ability to control certain factors, it represents an important first step in recognizing key determinants of mortality and improving the quality of care provided at this tertiary hospital. More work is needed to reduce delay in transfers to the ICU from emergency department and to reduce the rates of nosocomial infections, and to apply antimicrobial stewardship programs for patients with community-acquired or nosocomial infections. Finally, a clinical audit needs to be implemented in practice in Jordan and other developing countries.

## Figures and Tables

**Table 1:** Baseline characteristics of study participants by outcome at discharge.

		Mortality			
Characteristic	Overall, N = 1323 ^1^	Missing	No N = 861 ^1^	Yes N = 442 ^1^	*p*-Value ^2^
**Patient age**	65 (17)	0 (0%)	63 (18)	69 (15)	<0.001
**Patient gender**		0 (0%)			0.2
Female	608 (47%)		412 (68%)	196 (32%)	
Male	695 (53%)		449 (65%)	246 (35%)	
**Delay of admission**	18 (15)	0 (0%)	18 (15)	17 (15)	0.067
**Admission delay > 6 h**		0 (0%)			0.006
No	298 (23%)		177 (59%)	121 (41%)	
Yes	1005 (77%)		684 (68%)	321 (32%)	
**ICU stay duration**	160 (174)	0 (0%)	123 (133)	231 (218)	<0.001
**Admitted through ER**		0 (0%)			0.003
No	308 (24%)		182 (59%)	126 (41%)	
Yes	995 (76%)		679 (68%)	316 (32%)	
**Pneumonia**		0 (0%)			<0.001
CAP	165 (13%)		89 (54%)	76 (46%)	
HAP	150 (12%)		77 (51%)	73 (49%)	
No	988 (76%)		695 (70%)	293 (30%)	
**DM**		0 (0%)			0.8
No	642 (49%)		422 (66%)	220 (34%)	
Yes	661 (51%)		439 (66%)	222 (34%)	
**HTN**		0 (0%)			>0.9
No	519 (40%)		343 (66%)	176 (34%)	
Yes	784 (60%)		518 (66%)	266 (34%)	
**CKD**		0 (0%)			0.4
No	940 (72%)		614 (65%)	326 (35%)	
Yes	363 (28%)		247 (68%)	116 (32%)	
**Liver cirrhosis**		0 (0%)			>0.9
No	1239 (95%)		819 (66%)	420 (34%)	
Yes	64 (4.9%)		42 (66%)	22 (34%)	
**IHD**		0 (0%)			>0.9
No	985 (76%)		650 (66%)	335 (34%)	
Yes	318 (24%)		211 (66%)	107 (34%)	
**Heart Failure**		0 (0%)			0.6
No	980 (75%)		644 (66%)	336 (34%)	
Yes	323 (25%)		217 (67%)	106 (33%)	
**COPD/Asthma**		0 (0%)			0.041
No	1136 (87%)		739 (65%)	397 (35%)	
Yes	167 (13%)		122 (73%)	45 (27%)	
**OSA**		0 (0%)			0.026
No	1230 (94%)		804 (65%)	426 (35%)	
Yes	73 (5.6%)		57 (78%)	16 (22%)	
**GCS**	13.84 (2.73)	6 (0.5%)	14.38 (1.85)	12.78 (3.69)	<0.001
**On vasopressor**		0 (0%)			<0.001
No	1018 (78%)		727 (71%)	291 (29%)	
Yes	285 (22%)		134 (47%)	151 (53%)	
**Intubated**		0 (0%)			<0.001
No	1198 (92%)		837 (70%)	361 (30%)	
Yes	105 (8.1%)		24 (23%)	81 (77%)	
**Hb**	11.29 (2.81)	2 (0.2%)	11.52 (2.75)	10.84 (2.87)	<0.001
**Cr**	2.36 (2.94)	5 (0.4%)	2.46 (3.23)	2.16 (2.25)	0.8
**APACHE score**	16 (8)	419 (32%)	15 (7)	19 (8)	<0.001
**GCS score**		6 (0.5%)			<0.001
8 or more	1224 (94%)		839 (69%)	385 (31%)	
Less than 8	73 (5.6%)		19 (26%)	54 (74%)	
**Main diagnosis: Pneumonia**		0 (0%)			<0.001
No	1033 (79%)		723 (70%)	310 (30%)	
Yes	270 (21%)		138 (51%)	132 (49%)	
**Main diagnosis: Urosepsis**		0 (0%)			0.5
No	1165 (89%)		773 (66%)	392 (34%)	
Yes	138 (11%)		88 (64%)	50 (36%)	
**Main diagnosis: Urosepsis type**		0 (0%)			0.4
No	1165 (89%)		773 (66%)	392 (34%)	
Not catheter associated	72 (5.5%)		49 (68%)	23 (32%)	
Catheter associated	66 (5.1%)		39 (59%)	27 (41%)	
**Main diagnosis: AKI**		0 (0%)			0.2
No	1185 (91%)		777 (66%)	408 (34%)	
Yes	118 (9.1%)		84 (71%)	34 (29%)	
**Main diagnosis: RF Type 2**		0 (0%)			0.078
No	1206 (93%)		789 (65%)	417 (35%)	
Yes	97 (7.4%)		72 (74%)	25 (26%)	
**Main diagnosis: UGIB**		0 (0%)			0.057
No	1231 (94%)		806 (65%)	425 (35%)	
Yes	72 (5.5%)		55 (76%)	17 (24%)	
**Main diagnosis: Stroke**		0 (0%)			>0.9
No	1242 (95%)		821 (66%)	421 (34%)	
Yes	61 (4.7%)		40 (66%)	21 (34%)	
**Main diagnosis: NSTEMI**		0 (0%)			0.5
No	1267 (97%)		839 (66%)	428 (34%)	
Yes	36 (2.8%)		22 (61%)	14 (39%)	
**Main diagnosis: ADHF**		0 (0%)			0.4
No	1269 (97%)		841 (66%)	428 (34%)	
Yes	34 (2.6%)		20 (59%)	14 (41%)	
**2nd diagnosis: AKI**		0 (0%)			0.076
No	1195 (92%)		798 (67%)	397 (33%)	
Yes	108 (8.3%)		63 (58%)	45 (42%)	
**2nd diagnosis: Pneumonia**		0 (0%)			0.7
No	1259 (97%)		833 (66%)	426 (34%)	
Yes	44 (3.4%)		28 (64%)	16 (36%)	
**2nd diagnosis: Urosepsis**		0 (0%)			<0.001
No	1275 (98%)		851 (67%)	424 (33%)	
Yes	28 (2.1%)		10 (36%)	18 (64%)	
**2nd diagnosis: ADHF**		0 (0%)			0.7
No	1277 (98%)		843 (66%)	434 (34%)	
Yes	26 (2.0%)		18 (69%)	8 (31%)	
**2nd diagnosis: RF Type 2**		0 (0%)			0.6
No	1280 (98%)		847 (66%)	433 (34%)	
Yes	23 (1.8%)		14 (61%)	9 (39%)	
**Urosepsis: Primary or secondary**		0 (0%)			0.040
No	1137 (87%)		763 (67%)	374 (33%)	
Yes	166 (13%)		98 (59%)	68 (41%)	

^1^ Mean (SD); n (%). ^2^ Wilcoxon rank sum test; Pearson’s Chi-squared test.

**Table 2:** Predictors of mortality by APACHE score only while dropping age, GCS, and Cr.

Characteristic	OR ^1^	95% CI ^1^	*p*-Value
**ICU stay duration**	1.00	1.00, 1.00	<0.001
**Admitted through ER**			
No	—	—	
Yes	0.64	0.47, 0.89	0.007
**CKD**			
No	—	—	
Yes	0.78	0.57, 1.05	0.10
**COPD/Asthma**			
No	—	—	
Yes	0.60	0.39, 0.90	0.017
**On vasopressor**			
No	—	—	
Yes	2.36	1.71, 3.26	<0.001
**Intubated**			
No	—	—	
Yes	5.67	3.41, 9.69	<0.001
**Hb**	0.92	0.88, 0.97	0.003
**APACHE score**	1.06	1.03, 1.08	<0.001
**Main diagnosis: Pneumonia**			
No	—	—	
Yes	1.87	1.36, 2.57	<0.001
**Main diagnosis: Stroke**			
No	—	—	
Yes	1.75	0.92, 3.22	0.079
**Main diagnosis: NSTEMI**			
No	—	—	
Yes	1.85	0.85, 3.89	0.11
**2nd diagnosis: Urosepsis**			
No	—	—	
Yes	3.17	1.34, 7.75	0.009

^1^ OR = odds ratio, CI = confidence interval.

**Table 3:** Predictors of mortality without APACHE score.

Characteristic	OR ^1^	95% CI ^1^	*p*-Value
**Patient age**	1.02	1.01, 1.02	<0.001
**ICU stay duration**	1.00	1.00, 1.00	<0.001
**Admitted through ER**			
No	—	—	
Yes	0.61	0.44, 0.84	0.003
**COPD/Asthma**			
No	—	—	
Yes	0.66	0.43, 1.00	0.056
**GCS**	0.86	0.81, 0.91	<0.001
**On vasopressor**			
No	—	—	
Yes	2.41	1.75, 3.33	<0.001
**Intubated**			
No	—	—	
Yes	3.52	1.97, 6.40	<0.001
**Hb**	0.92	0.87, 0.96	<0.001
**Main diagnosis: Pneumonia**			
No	—	—	
Yes	1.65	1.21, 2.27	0.002
**2nd diagnosis: Urosepsis**			
No	—	—	
Yes	2.67	1.13, 6.55	0.027

^1^ OR = odds ratio, CI = confidence interval.

**Table 4:** Predictors of mortality with the APACHE score and all its contributing factors, including age, GCS, and Cr.

Characteristic	OR ^1^	95% CI ^1^	*p*-Value
**Patient age**	1.01	1.00, 1.02	0.003
**ICU stay duration**	1.00	1.00, 1.00	<0.001
**Admitted through ER**			
No	—	—	
Yes	0.62	0.45, 0.86	0.004
**COPD/Asthma**			
No	—	—	
Yes	0.62	0.40, 0.94	0.026
**GCS**	0.88	0.82, 0.93	<0.001
**On vasopressor**			
No	—	—	
Yes	2.30	1.66, 3.18	<0.001
**Intubated**			
No	—	—	
Yes	3.43	1.91, 6.25	<0.001
**Hb**	0.92	0.87, 0.97	0.001
**Cr**	0.96	0.91, 1.01	0.2
**APACHE score**	1.03	1.01, 1.06	0.018
**Main diagnosis: Pneumonia**			
No	—	—	
Yes	1.65	1.20, 2.26	0.002
**2nd diagnosis: Urosepsis**			
No	—	—	
Yes	2.64	1.12, 6.48	0.029

^1^ OR = odds ratio, CI = confidence interval.

## Data Availability

The data that support the findings of this study are not publicly available due to ethical restrictions, and they are available from the corresponding author upon reasonable request.

## Abbreviations

The following abbreviations are used in this manuscript:

ICU	Intensive Care Unit
APACHE	Acute Physiology and Chronic Health Evaluation
Emergency department	Accident and Emergency
GCS	Glasgow Coma Scale
AKI	Acute kidney injury
JUH	Jordan University Hospital
LOS	length of stay
HTN	Hypertension
DM	Diabetes mellitus
COPD	Chronic obstructive pulmonary disease
OSA	Obstructive sleep apnea
HAP	Hospital-acquired pneumonia
CAP	Community-acquired pneumonia
Hb	Hemoglobin
SD	Standard deviation
OR	Odds ratio
CI	Confidence interval
COVID-19	Coronavirus disease of 2019
AUROC	Area under the receiver operator characteristic curve

## Appendix A

**Table A1:** Baseline characteristics by route of admission through the Accident and Emergency department or not.

		Admitted Through ER			
Characteristic	Overall, N = 1323 ^1^	Missing	No N = 308 ^1^	Yes N = 995 ^1^	*p*-Value ^2^
**Patient age**	65 (17)	0 (0%)	64 (17)	65 (17)	0.2
**Patient gender**		0 (0%)			0.024
Female	608 (47%)		161 (26%)	447 (74%)	
Male	695 (53%)		147 (21%)	548 (79%)	
**Delay of admission**	18 (15)	0 (0%)	11 (11)	20 (16)	<0.001
**Admission delay > 6 h**		0 (0%)			<0.001
No	298 (23%)		135 (45%)	163 (55%)	
Yes	1005 (77%)		173 (17%)	832 (83%)	
**ICU stay duration**	160 (174)	0 (0%)	253 (213)	130 (149)	<0.001
**Pneumonia**		0 (0%)			0.061
CAP	165 (13%)		38 (23%)	127 (77%)	
HAP	150 (12%)		47 (31%)	103 (69%)	
No	988 (76%)		223 (23%)	765 (77%)	
**DM**		0 (0%)			0.14
No	642 (49%)		163 (25%)	479 (75%)	
Yes	661 (51%)		145 (22%)	516 (78%)	
**HTN**		0 (0%)			0.010
No	519 (40%)		142 (27%)	377 (73%)	
Yes	784 (60%)		166 (21%)	618 (79%)	
**CKD**		0 (0%)			0.6
No	940 (72%)		226 (24%)	714 (76%)	
Yes	363 (28%)		82 (23%)	281 (77%)	
**Liver cirrhosis**		0 (0%)			0.5
No	1239 (95%)		295 (24%)	944 (76%)	
Yes	64 (4.9%)		13 (20%)	51 (80%)	
**IHD**		0 (0%)			0.8
No	985 (76%)		231 (23%)	754 (77%)	
Yes	318 (24%)		77 (24%)	241 (76%)	
**Heart Failure**		0 (0%)			0.8
No	980 (75%)		233 (24%)	747 (76%)	
Yes	323 (25%)		75 (23%)	248 (77%)	
**COPD/Asthma**		0 (0%)			0.4
No	1136 (87%)		264 (23%)	872 (77%)	
Yes	167 (13%)		44 (26%)	123 (74%)	
**OSA**		0 (0%)			0.6
No	1230 (94%)		289 (23%)	941 (77%)	
Yes	73 (5.6%)		19 (26%)	54 (74%)	
**GCS**	13.84 (2.73)	6 (0.5%)	14.57 (1.75)	13.62 (2.93)	<0.001
**On vasopressor**		0 (0%)			<0.001
No	1018 (78%)		290 (28%)	728 (72%)	
Yes	285 (22%)		18 (6.3%)	267 (94%)	
**Intubated**		0 (0%)			0.005
No	1198 (92%)		295 (25%)	903 (75%)	
Yes	105 (8.1%)		13 (12%)	92 (88%)	
**Mortality**		0 (0%)			0.003
No	861 (66%)		182 (21%)	679 (79%)	
Yes	442 (34%)		126 (29%)	316 (71%)	
**Hb**	11.29 (2.81)	2 (0.2%)	11.22 (2.61)	11.31 (2.87)	0.5
**Cr**	2.36 (2.94)	5 (0.4%)	1.70 (1.76)	2.56 (3.19)	<0.001
**APACHE score**	16 (8)	419 (32%)	15 (8)	17 (7)	<0.001
**GCS score**		6 (0.5%)			0.002
8 or more	1224 (94%)		299 (24%)	925 (76%)	
Less than 8	73 (5.6%)		6 (8.2%)	67 (92%)	
**Main diagnosis: Pneumonia**		0 (0%)			0.10
No	1033 (79%)		234 (23%)	799 (77%)	
Yes	270 (21%)		74 (27%)	196 (73%)	
**Main diagnosis: Urosepsis**		0 (0%)			0.004
No	1165 (89%)		289 (25%)	876 (75%)	
Yes	138 (11%)		19 (14%)	119 (86%)	
**Main diagnosis: Urosepsis type**		0 (0%)			0.014
No	1165 (89%)		289 (25%)	876 (75%)	
Not catheter associated	72 (5.5%)		11 (15%)	61 (85%)	
Catheter associated	66 (5.1%)		8 (12%)	58 (88%)	
**Main diagnosis: AKI**		0 (0%)			<0.001
No	1185 (91%)		296 (25%)	889 (75%)	
Yes	118 (9.1%)		12 (10%)	106 (90%)	
**Main diagnosis: RF Type 2**		0 (0%)			0.024
No	1206 (93%)		276 (23%)	930 (77%)	
Yes	97 (7.4%)		32 (33%)	65 (67%)	
**Main diagnosis: UGIB**		0 (0%)			<0.001
No	1231 (94%)		303 (25%)	928 (75%)	
Yes	72 (5.5%)		5 (6.9%)	67 (93%)	
**Main diagnosis: Stroke**		0 (0%)			0.2
No	1242 (95%)		298 (24%)	944 (76%)	
Yes	61 (4.7%)		10 (16%)	51 (84%)	
**Main diagnosis: NSTEMI**		0 (0%)			0.010
No	1267 (97%)		306 (24%)	961 (76%)	
Yes	36 (2.8%)		2 (5.6%)	34 (94%)	
**Main diagnosis: ADHF**		0 (0%)			0.4
No	1269 (97%)		298 (23%)	971 (77%)	
Yes	34 (2.6%)		10 (29%)	24 (71%)	
**2nd diagnosis: AKI**		0 (0%)			0.2
No	1195 (92%)		277 (23%)	918 (77%)	
Yes	108 (8.3%)		31 (29%)	77 (71%)	
**2nd diagnosis: Pneumonia**		0 (0%)			0.9
No	1259 (97%)		298 (24%)	961 (76%)	
Yes	44 (3.4%)		10 (23%)	34 (77%)	
**2nd diagnosis: Urosepsis**		0 (0%)			0.2
No	1275 (98%)		304 (24%)	971 (76%)	
Yes	28 (2.1%)		4 (14%)	24 (86%)	
**2nd diagnosis: ADHF**		0 (0%)			0.053
No	1277 (98%)		306 (24%)	971 (76%)	
Yes	26 (2.0%)		2 (7.7%)	24 (92%)	
**2nd diagnosis: RF Type 2**		0 (0%)			0.078
No	1280 (98%)		299 (23%)	981 (77%)	
Yes	23 (1.8%)		9 (39%)	14 (61%)	
**Urosepsis: Primary or secondary**		0 (0%)			0.001
No	1137 (87%)		285 (25%)	852 (75%)	
Yes	166 (13%)		23 (14%)	143 (86%)	

^1^ Mean (SD); n (%). ^2^ Wilcoxon rank sum test; Pearson’s Chi-squared test.

**Table A2:** Performance summary of the three logistic regression models developed.

Metric	Model 1: APACHE Only	Model 2: No APACHE Score	Model 3: All Features
Accuracy	73.22%	74.06%	74.14%
Sensitivity	89.2%	88.97%	89.2%
Specificity	42.08%	45.02%	44.8%
AUROC	0.78	0.79	0.79
Brier score	0.17	0.17	0.17

## References

[1] Gopalan P.D., Pershad S. (2019). Decision-making in ICU—A systematic review of factors considered important by ICU clinician decision makers with regard to ICU triage decisions. J. Crit. Care.

[2] Tang H., Jin Z., Deng J., She Y., Zhong Y., Sun W., Ren Y., Cao N., Chen C. (2022). Development and validation of a deep learning model to predict the survival of patients in ICU. J. Am. Med. Inform. Assoc. JAMIA.

[3] Ohno-Machado L., Resnic F.S., Matheny M.E. (2006). PROGNOSIS IN CRITICAL CARE. Annu. Rev. Biomed. Eng..

[4] Groenland C.N.L., Termorshuizen F., Rietdijk W.J.R., van den Brule J., Dongelmans D.A., de Jonge E., de Lange E.W., de Smet A.M.G.A., de Keizer N.F., Weigel J.D. (2019). Emergency Department to ICU Time Is Associated with Hospital Mortality: A Registry Analysis of 14,788 Patients From Six University Hospitals in The Netherlands. Crit. Care Med..

[5] Parkhe M., Myles P.S., Leach D.S., Maclean A.V. (2002). Outcome of emergency department patients with delayed admission to an intensive care unit. Emerg. Med..

[6] Worku A., Haisch D., Parekh M., Sultan A., Shumet A., G/Selassie K., O’Donnell M., Binegdie A., Sherman C.B., Schluger N.W. (2024). Epidemiology and Outcomes of Critical Illness and Novel Predictors of Mortality in an Ethiopian Medical Intensive Care Unit. J. Intensive Care Med..

[7] Almansour I.M., Ahmad M.M., Alnaeem M.M. (2020). Characteristics, Mortality Rates, and Treatments Received in Last Few Days of Life for Patients Dying in Intensive Care Units: A Multicenter Study. Am. J. Hosp. Palliat. Care.

[8] Godinjak A., Iglica A., Rama A., Tančica I., Jusufović S., Ajanović A., Kukuljac A. (2016). Predictive value of SAPS II and APACHE II scoring systems for patient outcome in a medical intensive care unit. Acta Medica Acad..

[9] Knaus W.A., Draper E.A., Wagner D.P., Zimmerman J.E. (1985). APACHE II: A severity of disease classification system. Crit. Care Med..

[10] Quintairos A., Pilcher D., Salluh J.I.F. (2023). ICU scoring systems. Intensive Care Med..

[11] Lloyd-Thomas A.R., Wright I., Lister T.A., Hinds C.J. (1988). Prognosis of patients receiving intensive care for lifethreatening medical complications of haematological malignancy. Br. Med. J. Clin. Res. Ed..

[12] Ludwigs U., Hulting J. (1995). Acute Physiology and Chronic Health Evaluation II scoring system in acute myocardial infarction: A prospective validation study. Crit Care Med..

[13] Maher E.R., Robinson K.N., Scoble J.E., Farrimond J.G., Browne D.R.G., Sweny P., Moorhead J.F. (1989). Prognosis of critically-ill patients with acute renal failure: APACHE II score and other predictive factors. QJM.

[14] Edwards A.T., Ng K.J., Shandall A.A., Price-Thomas J.M. (1991). Experience with the APACHE II severity of disease scoring system in predicting outcome in a surgical intensive therapy unit. J. R. Coll. Surg. Edinb..

[15] Headley J., Theriault R., Smith T.L. (1992). Independent validation of APACHE II severity of illness score for predicting mortality in patients with breast cancer admitted to the intensive care unit. Cancer.

[16] Song X., Liu X.Y., Wang H.R., Guo X.Y., Kashani K.B., Ma P.L. (2021). Association between anemia and ICU outcomes. Chin. Med.J..

[17] Definition of Boarded Patient. https://www.acep.org/patient-care/policy-statements/definition-of-boarded-patient.

[18] Angotti L.B., Richards J.B., Fisher D.F., Sankoff J.D., Seigel T.A., Al Ashry H.S., Wilcox S.R. (2017). Duration of Mechanical Ventilation in the Emergency Department. West. J. Emerg. Med..

[19] Chalfin D.B., Trzeciak S., Likourezos A., Baumann B.M., Dellinger R.P., DELAY-ED study group (2007). Impact of delayed transfer of critically ill patients from the emergency department to the intensive care unit. Crit. Care Med..

[20] Goldhill D.R., McNarry A.F., Hadjianastassiou V.G., Tekkis P.P. (2004). The longer patients are in hospital before Intensive Care admission the higher their mortality. Intensive Care Med..

[21] Agustin M., Price L.L., Andoh-Duku A., LaCamera P. (2017). Impact of Delayed Admission to the Intensive Care Unit from the Emergency Department upon Sepsis Outcomes and Sepsis Protocol Compliance. Crit. Care Res. Pract..

[22] Cardoso L.T., Grion C.M., Matsuo T., Anami E.H., Kauss I.A., Seko L., Bonametti A.M. (2011). Impact of delayed admission to intensive care units on mortality of critically ill patients: A cohort study. Crit. Care.

[23] Mathews K.S., Durst M.S., Vargas-Torres C., Olson A.D., Mazumdar M., Richardson L.D. (2018). Effect of Emergency Department and ICU Occupancy on Admission Decisions and Outcomes for Critically Ill Patients. Crit. Care Med..

[24] Singer A.J., Thode H.C., Viccellio P., Pines J.M. (2011). The association between length of emergency department boarding and mortality. Acad. Emerg. Med. Off. J. Soc. Acad. Emerg. Med..

[25] Rincon F., Mayer S.A., Rivolta J., Stillman J., Boden-Albala B., Elkind M.S.V., Marshall R., Chong J.Y. (2010). Impact of delayed transfer of critically ill stroke patients from the Emergency Department to the Neuro-ICU. Neurocrit. Care.

[26] Dhand N., Khatkar M. (2014). Sample Size Calculator for Estimating a Proportion. https://statulator.com/SampleSize/ss1P.html.

[27] Demass T.B., Guadie A.G., Mengistu T.B., Belay Z.A., Melese A.A., Berneh A.A., Mihret L.G., Wagaye F.E., Bantie G.M. (2023). The magnitude of mortality and its predictors among adult patients admitted to the Intensive care unit in Amhara Regional State, Northwest Ethiopia. Sci. Rep..

[28] Unal A.U., Kostek O., Takir M., Caklili O., Uzunlulu M., Oguz A. (2015). Prognosis of patients in a medical intensive care unit. North. Clin. Istanb..

[29] Capuzzo M., Volta C., Tassinati T., Moreno R., Valentin A., Guidet B., Iapichino G., Martin C., Perneger T., Combescure C. (2014). Hospital mortality of adults admitted to Intensive Care Units in hospitals with and without Intermediate Care Units: A multicentre European cohort study. Crit. Care.

[30] Mayyas F., Tashtoush M., Tashtoush Z. (2023). Predictors of intensive care unit length of stay and mortality among unvaccinated COVID-19 patients in Jordan. Infect. Prev. Pract..

[31] Al Oweidat K., Al-Amer R., Saleh M.Y., Albtoosh A.S., Toubasi A.A., Ribie M.K., Hasuneh M.M., Alfaqheri D.L., Alshurafa A.H., Ribie M. (2023). Mortality, Intensive Care Unit Admission, and Intubation among Hospitalized Patients with COVID-19: A One-Year Retrospective Study in Jordan. J. Clin. Med..

[32] Kiekkas P., Tzenalis A., Gklava V., Stefanopoulos N., Voyagis G., Aretha D. (2022). Delayed Admission to the Intensive Care Unit and Mortality of Critically Ill Adults: Systematic Review and Meta-analysis. BioMed Res. Int..

[33] Hope A.A., Gong M.N., Guerra C., Wunsch H. (2015). Frailty Before Critical Illness and Mortality for Elderly Medicare Beneficiaries. J. Am. Geriatr. Soc..

[34] McNelly A.S., Rawal J., Shrikrishna D., Hopkinson N.S., Moxham J., Harridge S.D., Hart N., Montgomery H.E., Puthucheary Z.A. (2016). An Exploratory Study of Long-Term Outcome Measures in Critical Illness Survivors: Construct Validity of Physical Activity, Frailty, and Health-Related Quality of Life Measures. Crit. Care Med..

[35] Bastos P.G., Sun X., Wagner D.P., Wu A.W., Knaus W.A. (1993). Glasgow Coma Scale score in the evaluation of outcome in the intensive care unit: Findings from the Acute Physiology and Chronic Health Evaluation III study. Crit. Care Med..

[36] Belletti A., Castro M.L., Silvetti S., Greco T., Biondi-Zoccai G., Pasin L., Zangrillo A., Landoni G. (2015). The Effect of inotropes and vasopressors on mortality: A meta-analysis of randomized clinical trials. BJA Br. J. Anaesth..

[37] Salacup G., Lo K.B., Gul F., Peterson E., De Joy R., Bhargav R., Pelayo J., Albano J., Azmaiparashvili Z., Benzaquen S. (2021). Characteristics and clinical outcomes of COVID-19 patients in an underserved-inner city population: A single tertiary center cohort. J. Med. Virol..

[38] Jung S.M., Kim Y.J., Ryoo S.M., Kim W.Y. (2019). Relationship between low hemoglobin levels and mortality in patients with septic shock. Acute Crit. Care.

[39] Tezcan B., Kosovali B.D., Can M., Demirbag A.E., Yavuz A., Mutlu N.M. (2023). The Relationship Between Mortality and Hemoglobin Levels in Intensive Care Patients with Acute Respiratory Distress Syndrome. J. Crit. Intensive Care.

[40] Thomas J., Jensen L., Nahirniak S., Gibney R.T.N. (2010). Anemia and blood transfusion practices in the critically ill: A prospective cohort review. Heart Lung J. Crit. Care.

[41] Hajjar L.A., Auler Junior J.O.C., Santos L., Galas F. (2007). Blood tranfusion in critically ill patients: State of the art. Clinics.

